# Interpretable radiomics signature of the basal ganglia-thalamic circuitry for predicting PD-MCI: a multi-center study with external validation

**DOI:** 10.1186/s12880-026-02668-7

**Published:** 2026-08-12

**Authors:** Yongjie Jian, Hui Shi, Tao Hu, Shengyi Zhou, Xiaojuan He, Di Tao, Hongcai Du, Jie Zhou, Yongping Yao, Zhenyu Shu

**Affiliations:** 1 Department of Radiology, Affiliated Hospital of Sichuan Nursing Vocational College, The Third People’s Hospital of Sichuan Province, Chengdu, Sichuan China; 2 Neuroscience Center, Affiliated Hospital of Sichuan Nursing Vocational College, The Third People’s Hospital of Sichuan Province, Chengdu, Sichuan China; 3 School of Pharmacy, Sichuan Nursing Vocational College, Chengdu, Sichuan China; 4 Department of Radiology, Zhejiang Provincial People’s Hospital, People’s Hospital of Hangzhou Medical College, Hangzhou, Zhejiang China

**Keywords:** Parkinson’s disease, Magnetic resonance imaging, Radiomics, Cognitive dysfunction

## Abstract

**Background:**

To develop and validate a T1-weighted imaging (T1WI)-based radiomic signature and integrate it with clinical features to construct a combined model for the early identification of cognitive decline in Parkinson’s disease (PD).

**Methods:**

We retrospectively analyzed data from 233 PD patients, including 182 from the Parkinson’s Progression Markers Initiative (PPMI) and 51 from the National Alzheimer’s Coordinating Center (NACC). Based on baseline Montreal Cognitive Assessment (MoCA) scores, patients were classified into those with normal cognition (PD-NC) and those with mild cognitive impairment (PD-MCI). The PPMI cohort was randomly divided into training (*n* = 127) and test (*n* = 55) sets at a 7:3 ratio, while the NACC cohort served as an external validation set (*n* = 51). Radiomic features were extracted from T1WI of the bilateral basal ganglia (globus pallidus, putamen) and bilateral thalamus. After dimensionality reduction, a radiomic model was constructed. Independent clinical predictors were identified and integrated with the radiomic signature to establish a combined model. Model performance was evaluated using receiver operating characteristic (ROC) curves, confusion matrices, and F1-scores. SHapley Additive exPlanations (SHAP) was employed to interpret model outputs.

**Results:**

The radiomic model achieved area under the curve (AUC) values of 0.809, 0.777, and 0.776 in the training, test, and external validation sets, respectively. Multivariable regression identified the Geriatric Depression Scale (GDS) score (odds ratio [OR] = 1.41, *P* = 0.005) and resting tremor (OR = 4.11, *P* = 0.036) as independent predictors. The combined model incorporating these clinical factors with the radiomic signature yielded significantly improved AUCs of 0.848 (95% CI: 0.774–0.906), 0.796 (95% CI: 0.666–0.893), and 0.812 (95% CI: 0.628–0.925) across the three datasets, outperforming either model alone. Among the 13 retained radiomic features, texture features were predominant (69.2%). SHAP analysis confirmed that the Rad-score was the most influential predictor.

**Conclusion:**

The combined model integrating T1WI-based radiomic features from the bilateral basal ganglia (globus pallidus, putamen)–thalamus with clinical characteristics demonstrates robust predictive capability for cognitive decline in PD. This approach offers valuable support for early intervention and precision management in clinical practice.

**Supplementary Information:**

The online version contains supplementary material available at https://doi.org/10.1186/s12880-026-02668-7.

## Background

Parkinson’s disease (PD), the second most common neurodegenerative disorder [1], is projected to affect approximately 25.2 million individuals by 2050, representing a 112% increase from 2021 levels [2]. The core neuropathology of PD is defined by the toxic accumulation of misfolded α-synuclein in the cytoplasm, forming Lewy bodies that drive the progressive loss of dopaminergic neurons [3]. Dysfunction within the basal ganglia circuitry is a key driver of both motor and non-motor symptoms [4]. Notably, this pathological process is closely linked to key nuclei within this circuit, particularly the globus pallidus (GP) and the putamen (PUT) [5, 6]. While the thalamus (TH) is largely spared from direct α-synuclein deposition, it serves as a critical downstream hub. Here, the loss of dopaminergic input leads to neurochemical imbalances and disrupts functional connectivity, significantly exacerbating the disease course [7].

Cognitive impairment represents one of the most prominent non-motor symptoms of PD [8], with approximately one-third of patients exhibiting mild cognitive impairment (MCI) at the early stage of the disease [9]. As the disease progresses, about 50% of these patients with early cognitive deficits will develop Parkinson’s disease dementia (PD-D) within 10–15 years [10]. Importantly, PD-MCI itself serves as a significant risk marker predicting progression to PD-D [11]. However, the exact pathophysiological mechanisms underlying PD-MCI remain unclear [12]. Accordingly, identifying biomarkers that reflect the pathological process for early detection of PD-MCI is crucial for formulating intervention strategies aimed at delaying the onset of dementia [13].

In the field of neuroimaging, magnetic resonance imaging (MRI), owing to its non-invasive nature and absence of ionizing radiation, has become an indispensable tool for the diagnosis and staging of PD [14]. In routine clinical practice, conventional structural MRI (sMRI) remains the mainstream modality [15]; however, subtle structural alterations in the brains of early-stage PD patients are often indiscernible to the naked eye [16, 17]. In recent years, advanced MRI sequences, such as neuromelanin-sensitive MRI (NM-MRI) [18], in conjunction with molecular imaging techniques like positron emission tomography (PET) [19], have been leveraged in PD diagnosis and mechanistic research. Nevertheless, the widespread clinical adoption of these technologies is constrained by their complex imaging principles and relatively high costs.

Radiomics, initially developed in oncology [20, 21] to extract high-throughput features for deciphering disease mechanisms, has since demonstrated distinct value in neurodegenerative disorders by capturing pre-symptomatic pathological changes. It offers a novel perspective for research breakthroughs by sensitively detecting subtle brain changes associated with pathological progression using sMRI data [22]. Our previous studies have demonstrated that whole-brain gray and white matter radiomic features derived from T1-weighted imaging (T1WI) can effectively predict the trajectory of cognitive decline in early-stage PD patients prior to the manifestation of clinical cognitive symptoms [23]. However, studies specifically analyzing key nuclei within the basal ganglia and the TH using T1WI remain scarce. Furthermore, while whole-brain radiomic approaches (including our own prior work [23]) provide valuable phenotypic insights, they often encounter the ‘curse of dimensionality’ and may dilute subtle, region-specific textural alterations within heterogeneous gray and white matter segments. Supporting this node-specific hypothesis, recent work has reclassified PD as a disorder of the somato-cognitive action network (SCAN) [24], highlighting that cognitive decline is driven by focal dysfunction within critical circuits.

Therefore, this study aimed to explore T1WI-derived radiomic features from specific basal ganglia nuclei (i.e., the GP and PUT) and the TH, and to construct a combined predictive model integrating these imaging features with clinical variables. We further evaluated the model’s utility in identifying PD-MCI at the early disease stage, thereby providing an objective basis for timely clinical intervention.

## Methods

### Participants

**Fig. 1:**
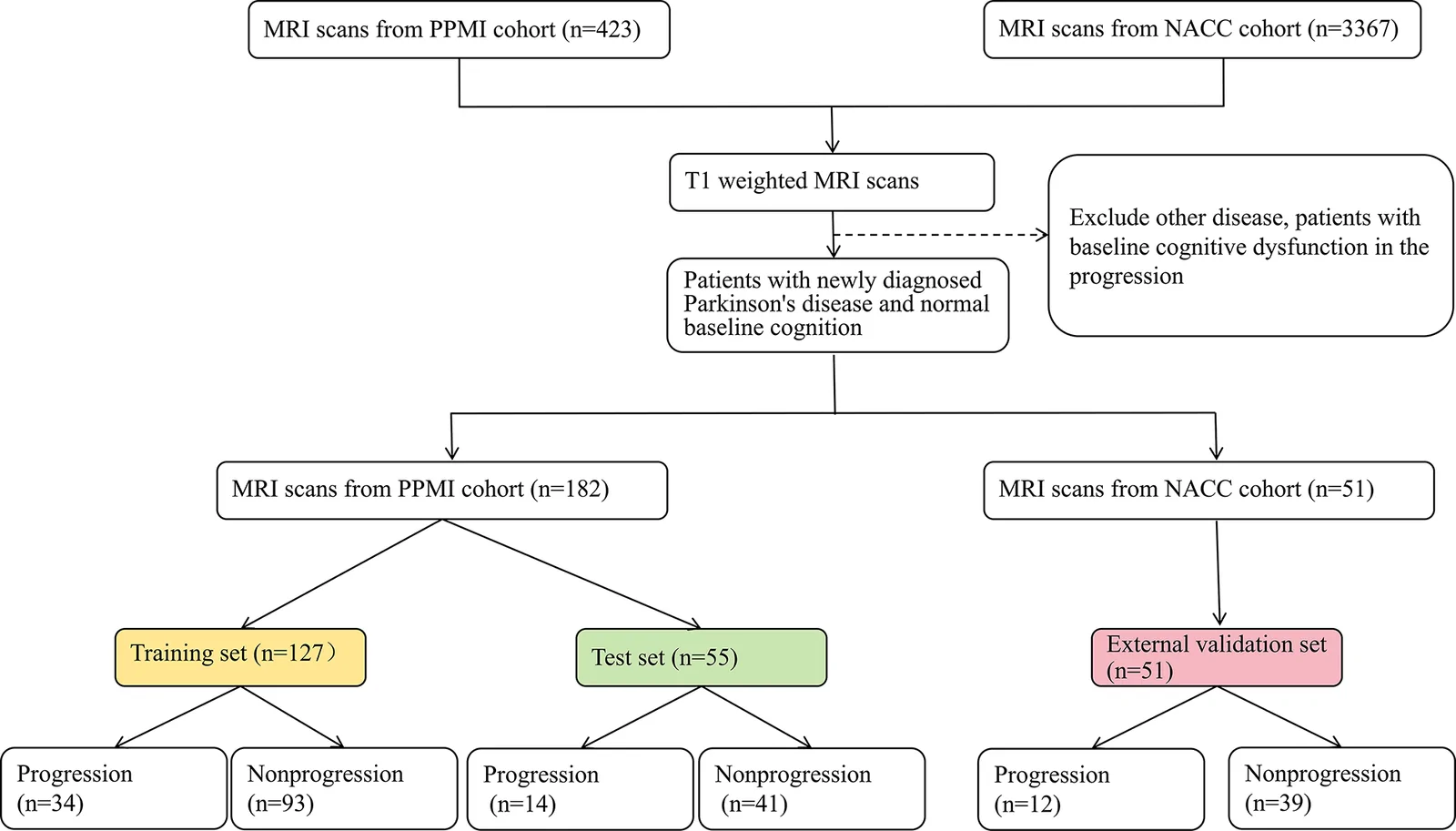
Flowchart of patient enrollment for the present study

This study was conducted in accordance with the ethical principles of the Declaration of Helsinki. We retrospectively included data from patients with PD enrolled in two large-scale, publicly available databases: the Parkinson’s Progression Markers Initiative (PPMI; https://www.ppmi-info.org*)* [25] and the National Alzheimer’s Coordinating Center (NACC; https://naccdata.org*)* [26]. The PPMI is a multicenter observational study designed to identify clinical, imaging, and biospecimen biomarkers of PD progression. The NACC maintains a standardized, longitudinal database, including patients with PD, of clinical, neuroimaging, and neuropathological data collected from Alzheimer’s Disease Research Centers across the United States. Ethical approval for the PPMI and NACC studies was obtained from the Institutional Review Boards (IRBs) of all participating sites, and written informed consent was obtained from all participants prior to enrollment. Details regarding the ethical oversight of these datasets are available on the respective websites. The current study was approved by the Ethics Committee of the Third People’s Hospital of Sichuan Province (Approval No. KY-2026-0105). The inclusion criteria were as follows: (1) Treatment-naïve PD patients; (2) Availability of baseline T1WI and complete clinical data; (3) Adequate image quality for radiomic analysis (i.e., absence of severe artifacts). The exclusion criteria included: (1) Failure to read automated segmentation result files; (2) Substantial deviations in automated segmentation results; (3) Comorbid neurodegenerative diseases or severe organic lesions. PD-MCI was defined as an education-adjusted Montreal Cognitive Assessment (MoCA) score ≤ 25, and PD-NC as ≥ 26 [27]. This binary classification served as the outcome variable for radiomics modeling across the multi-center cohorts. Based on these criteria, a total of 233 PD patients were ultimately enrolled. For the PPMI cohort (*n* = 182), 48 patients progressed to PD-MCI over a 4-year follow-up period (progressor group), while 134 remained stable (non-progressor group). This cohort was randomly divided into a training set (*n* = 127) and a test set (*n* = 55) at a ratio of 7:3. The external validation set comprised 51 patients from the NACC cohort, including 12 progressors and 39 non-progressors. The detailed participant screening flowchart is presented in Fig. 1.

### T1-weighted MRI Preprocessing

All imaging data were acquired using 1.5T or 3.0T MRI scanners. The T1WI protocol consisted of the following parameters: slice thickness = 1 mm, voxel resolution = 1 × 1 × 1 mm³, matrix = 256 × 256, and field of view (FOV) = 256 × 256 mm. Other sequence parameters (repetition time [TR], echo time [TE], and flip angle [FA]) varied according to the standard protocols of the respective scanners. Detailed MRI parameters and imaging protocols are documented in the PPMI and NACC MRI operation manuals, which specify standardization requirements for scanner models, scanning parameters, and imaging protocols. Given the multi-center origin of the imaging data, all T1WI images underwent a standardized preprocessing pipeline: (1) image denoising and N4 bias field correction; (2) removal of non-brain tissues (e.g., scalp and skull); (3) non-linear registration to the template of the MNI152 standard brain space; (4) isotropic resampling to a voxel spacing of 1 × 1 × 1 mm using third-order B-spline interpolation to standardize the voxel space, thereby ensuring that texture matrices were extracted on a consistent spatial grid across all scanners; and (5) segmentation of regions of interest (ROIs) using the Automated Anatomical Labeling Atlas 3 (AAL3) [28]. Two board-certified radiologists visually inspected the segmentation results using 3D Slicer (version 5.6.2, https://www.slicer.org) and manually corrected them when necessary. Manual corrections were performed if (1) the ROI included non-target tissues (e.g., adjacent white matter) or (2) the ROI failed to cover the target region. To comprehensively assess segmentation reproducibility, both geometric overlap and quantitative feature stability were evaluated. Geometric consistency was measured using the Dice similarity coefficient (DSC > 0.85 indicating satisfactory agreement). Furthermore, intra- and inter-observer reliability of the extracted radiomic features was assessed using the ICC based on a two-way random-effects model, single measures, and absolute agreement, ICC(2,1). Radiologists were blinded to the clinical data during the segmentation process.

### Radiomic feature extraction

Based on the PyRadiomics package (version 3.1.0; conforming to the Image Biomarker Standardization Initiative [IBSI] guidelines [29]), 107 quantitative radiomic features were extracted from each ROI (bilateral GP, PUT, and TH on T1WI sequences). These features comprised first-order statistics (18 features; e.g., mean, variance, skewness), texture features (75 features; derived from neighboring gray tone difference matrix [NGTDM], gray-level co-occurrence matrix [GLCM], gray-level dependence matrix [GLDM], Gray-Level Size Zone Matrix [GLSZM], and gray-level run-length matrix [GLRLM]), and shape-based features (14 features; e.g., volume, surface area, sphericity). Thus, a total of 642 features (6 ROIs × 107 features) were extracted per subject. Detailed feature information is provided in Table S1 of the Supplementary Material.

### Feature selection and radiomic signature construction

To avoid overfitting and ensure model generalizability, feature selection and radiomic signature construction were performed strictly within the training cohort. Initially, Z-score normalization was applied to the radiomic features, with the mean and standard deviation calculated exclusively from the training set and subsequently applied to the test and external validation sets. Subsequently, the minimum redundancy maximum relevance (mRMR) algorithm was employed to select the top 100 features most correlated with PD-MCI status. Least Absolute Shrinkage and Selection Operator (LASSO) logistic regression was then applied for further dimensionality reduction. The optimal regularization parameter (λ = 0.03) was determined via 10-fold cross-validation conducted exclusively within the training set. To address the inherent class imbalance (progressors: 26.7%), class weights were incorporated during model fitting, assigning higher weights to the minority (progressor) class to increase the penalty for misclassification. Features with non-zero coefficients at the optimal λ were retained, resulting in 13 radiomic features used to construct the Rad-score. The weights of these 13 features and the fixed λ were frozen upon completion of the training phase and uniformly applied to calculate Rad-scores for the test and external validation sets without any refitting or re-selection.

### Development and validation of the combined model

Thirteen clinical variables were initially screened for potential predictors using univariate logistic regression, followed by multivariate logistic regression to identify independent predictors. A combined model was constructed using logistic regression, incorporating both the Rad-score and identified clinical independent predictors (GDS score and resting tremor). Based on this model, a visual nomogram was established to facilitate individualized risk assessment. The predictive performance of the models was evaluated using receiver operating characteristic (ROC) curves, sensitivity, specificity, calibration curves, and decision curve analysis (DCA). Furthermore, SHapley Additive exPlanations (SHAP) was utilized to interpret the optimal model developed in the training set to evaluate the contribution of each feature to the model predictions.

**Fig. 2:**
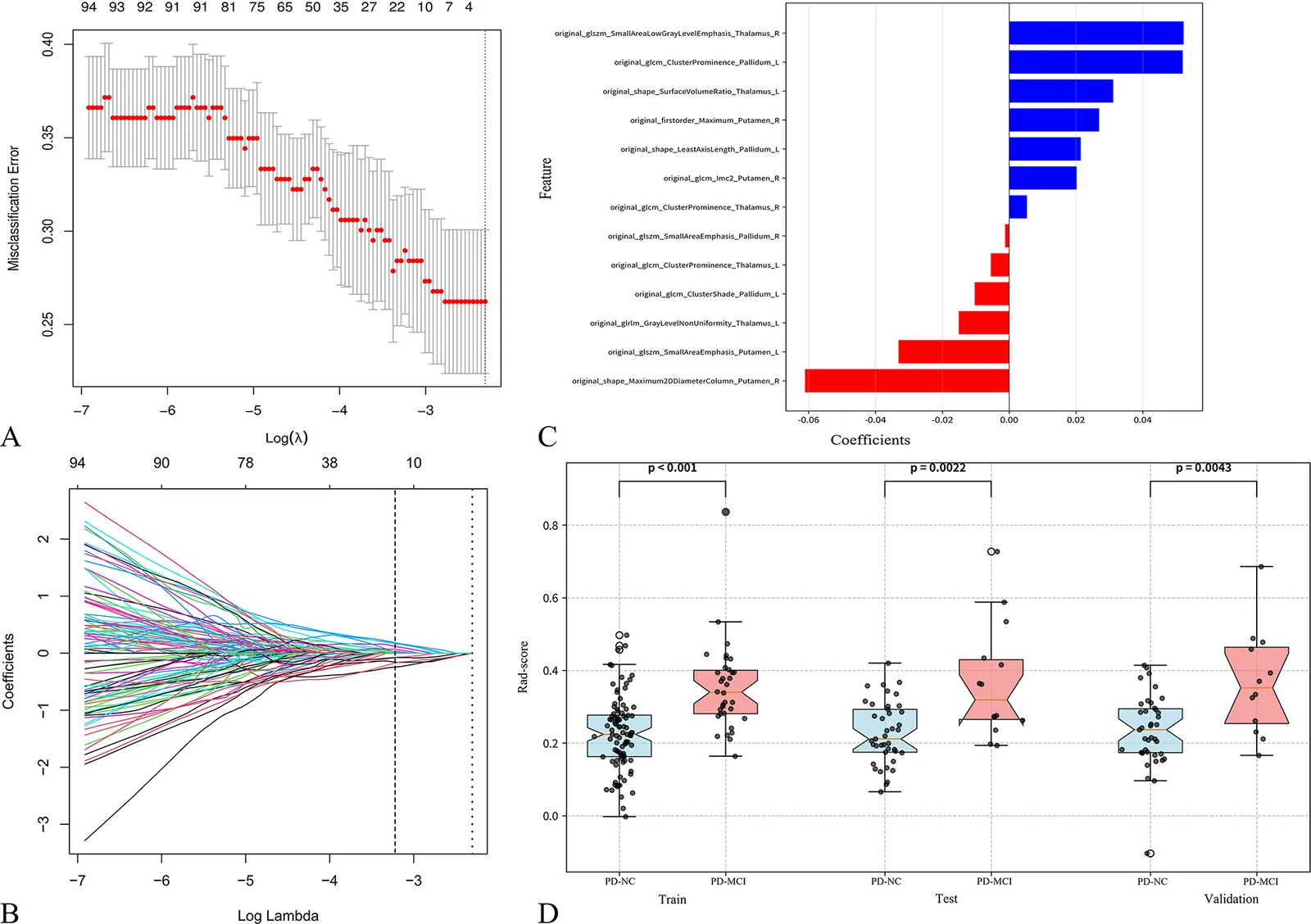
Summary of feature selection and Wilcoxon test results in the radiomics model. (**A**) Tracking plot of texture feature coefficients fitted by the LASSO regression. (**B**) The process for determining the penalty parameter (λ) via 10-fold cross-validation. (**C**) Remaining features after the selection process. In this panel, the y-axis represents each radiomics feature, and the x-axis represents their corresponding coefficients. (**D**) Results of the Wilcoxon test in the training, test, and external validation sets

### Statistical analysis

Statistical analyses were performed using SPSS software (version 25.0) and MedCalc. The Shapiro-Wilk test was used to assess the normality of continuous variables. Normally distributed data were expressed as mean ± standard deviation, whereas non-normally distributed data were presented as median (interquartile range, IQR). Categorical variables were described as frequencies and percentages. Group comparisons were assessed using the chi-square test or Fisher’s exact test where appropriate. *P*-values were calculated using the chi-square test for categorical variables and Student’s t-test for continuous variables, with statistical significance set at *P* < 0.05. All *P*-values are presented rounded to three decimal places. Multivariate analysis was conducted using logistic regression, adhering to this significance threshold.

## Results

### Clinical characteristics

A total of 233 PD patients were enrolled in this study, and their baseline clinical characteristics are summarized in Table 1. The PPMI dataset was randomly divided into a training set (*n* = 127) and a test set (*n* = 55) at a 7:3 ratio. Within the PPMI cohort, 48 patients (26.4%) progressed to PD-MCI during the 4-year follow-up period, while 134 patients (73.6%) remained cognitively stable (non-progressors). The NACC dataset served as an external validation set, including 51 PD patients, of whom 12 (23.5%) progressed to PD-MCI, and 39 (76.5%) remained stable. Univariate analysis revealed significant differences in age (*P* < 0.001), MoCA score (*P* < 0.001), GDS score (*P* = 0.005), and years of education (*P* < 0.001) between progressors and non-progressors in the training set.

**Table 1:** Baseline characteristics of the included studies

Characteristics		PPMI							NACC		
		Training set (n=127)			Test set (n = 55)				External validation set (n = 51)		
		Nonprogression	Progression (n=34)	P value	Nonprogression	Progression (n=14)	P value		Nonprogression (n=39)	Progression (n=12)	P value
		(n=93)			(n=41)						
Sex (n, %)	Female	59 (63.4%)	24 (70.6%)	0.59	24 (58.5%)	10 (71.4%)		0.59	23 (59.0%)	7 (58.3%)	1
	Male	34 (36.6%)	10 (29.4%)		17 (41.5%)	4 (28.6%)			16 (41.0%)	5 (41.7%)	
Age (years)		65.20 (58.43-72.08)	68.50 (61.16-75.50)	< 0.001	63.51 (59.59-69.15)	68.89 (61.44-73.37)		0.139	68.00 (65.00-72.00)	73.50 (67.00-79.00)	0.095
MoCA		29.00 (28.00-30.00)	28.00 (26.00-29.00)	< 0.001	28.00 (27.00-29.00)	27.00 (26.25-28.00)		0.094	28.00 (27.00-29.00)	26.50 (26.00-28.25)	0.095
GDS		1 (0.00-2.00)	2.00 (1.00-3.00)	0.005	1.00 (0.00-2.00)	2.00 (1.00-3.00)		0.223	2.00 (1.00-3.00)	3.00 (0.75-4.00)	0.235
Educyrs		12.00 (10.00-16.00)	16.00 (12.00-18.00)	< 0.001	16.00 (14.00-18.00)	16.00 (15.00-17.50)		0.71	18.00 (16.00-18.00)	17.00 (15.50-19.25)	0.972
Resting Tremor (n, %)	No	23 (24.7%)	3 (8.8%)	0.086	9 (22.0%)	1 (7.1%)		0.423	22 (56.4%)	5 (41.7%)	0.573
	Yes	70 (75.3%)	31 (91.2%)		32 (78.0%)	13 (92.9%)			17 (43.6%)	7 (58.3%)	
Rigidity (n, %)	No	26 (28.0%)	7 (20.6%)	0.542	9 (22.0%)	4 (28.6%)		0.719	NA^a^	NA^a^	NA
	Yes	67 (72.0%)	27 (79.4%)		32 (78.0%)	10 (71.4%)					
Bradykinesia (n, %)	No	15 (16.1%)	7 (20.6%)	0.747	5 (12.2%)	1 (7.1%)		1	15 (38.5%)	4 (33.3%)	1
	Yes	78 (83.9%)	27 (79.4%)		36 (87.8%)	13 (92.9%)			24 (61.5%)	8 (66.7%)	
Postural Instability (n, %)	No	8 (8.6%)	6 (17.6%)	0.199	37 (90.2%)	14 (100.0%)		0.562	19 (48.7%)	6 (50.0%)	1
	Yes	85 (91.4%)	28 (82.4%)		4 (9.8%)	0			20 (51.3%)	6 (50.0%)	
Other (n, %)	No	18 (19.4%)	4 (11.8%)	0.462	33 (80.5%)	12 (85.7%)		1	NA^a^	NA^a^	NA
	Yes	75 (80.6%)	30 (88.2%)		8 (19.5%)	2 (14.3%)					
H-Y (n, %)	No	53 (57.0%)	14 (41.2%)	0.168	15 (36.6%)	5 (35.7%)		1	NA^a^	NA^a^	NA
	Yes	40 (43.0%)	20 (58.8%)		26 (63.4%)	9 (64.3%)					
ESS (n, %)	No	79 (84.9%)	31 (91.2%)	0.557	29 (70.7%)	12 (85.7%)		0.478	NA^a^	NA^a^	NA
	Yes	14 (15.1%)	3 (8.8%)		12 (29.3%)	2 (14.3%)					
RBD (n, %)	No	67 (72.0%)	21 (61.8%)	0.371	26 (63.4%)	6 (42.9%)		0.302	28 (71.8%)	9 (75.0%)	1
	Yes	26 (28.0%)	13 (38.2%)		15 (36.6%)	8 (57.1%)			11 (28.2%)	3 (25.0%)	

Note. Age (year), Age at Enrollment; Educyrs, Years of Education; MoCA, MoCA score (adjusted for education); H-Y, Categorical Hoehn & Yahr; ESS, Categorical Epworth Sleepiness Scale Score; RBD, Categorical REM Sleep Behavior Disorder; GDS, Categorical Geriatric Depression Scale; NA = not available; ^a^, not available for all NACC study participants

### Radiomic feature selection and performance

**Fig. 3:**
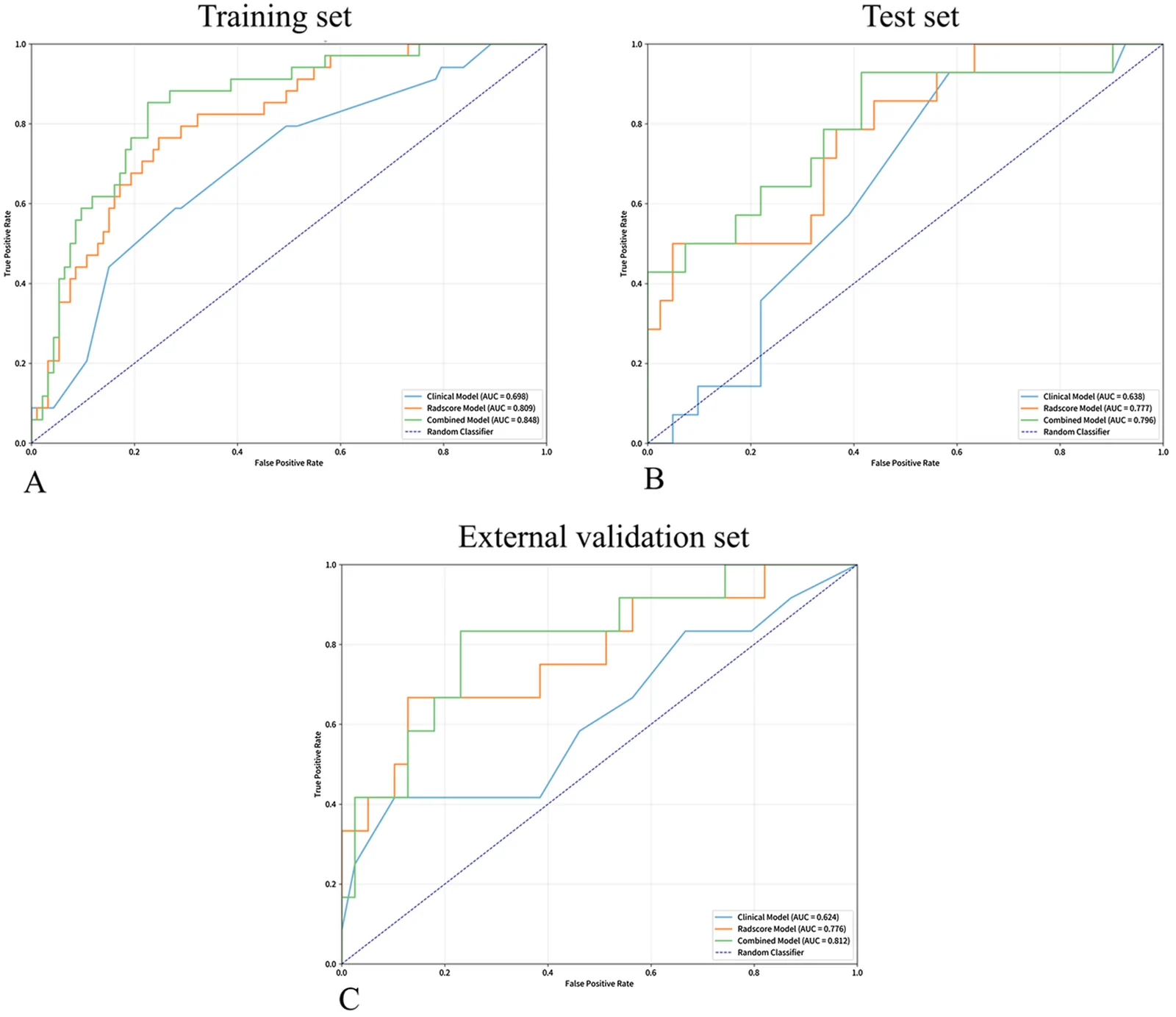
ROC curves of the combined model, radiomics model, and clinical model. **A**, **B** and **C** show the ROC curves of the combined model, radiomics model, and clinical model respectively in the training set, test set and external validation sets

Following feature selection using the mRMR algorithm, LASSO regression (10-fold cross-validation, λ = 0.03) retained 13 features with non-zero coefficients (Fig. 2A, B, C) to construct the Rad-score. Segmentation reproducibility was quantitatively assessed across the six ROIs. The mean Dice similarity coefficient (DSC ± SD) was 0.88 ± 0.04 for the bilateral GP, 0.86 ± 0.05 for the bilateral PUT, and 0.91 ± 0.03 for the bilateral TH, all exceeding the pre-defined threshold of 0.85. Furthermore, intra-observer ICC ranged from 0.94 to 0.96, and inter-observer ICC ranged from 0.89 to 0.92 across the six ROIs, indicating excellent quantitative stability. The calculation formula for the Rad-score is provided in Figure S1 of the Supplementary Material. The Rad-score demonstrated robust discriminative ability, yielding area under the curve (AUC) values of 0.809 (95% CI: 0.730–0.874), 0.777 (95% CI: 0.645–0.878), and 0.776 (95% CI: 0.637–0.881) in the training, test, and external validation sets, respectively (Fig. 3). The Wilcoxon rank-sum test indicated that Rad-score distribution differed significantly across the three datasets (training set: *P* < 0.001; test set: *P* = 0.002; external validation set: *P* = 0.004) (Fig. 2D), further validating the predictive value of the radiomic features.

### Development of the combined clinical-radiomic model

**Fig. 4:**
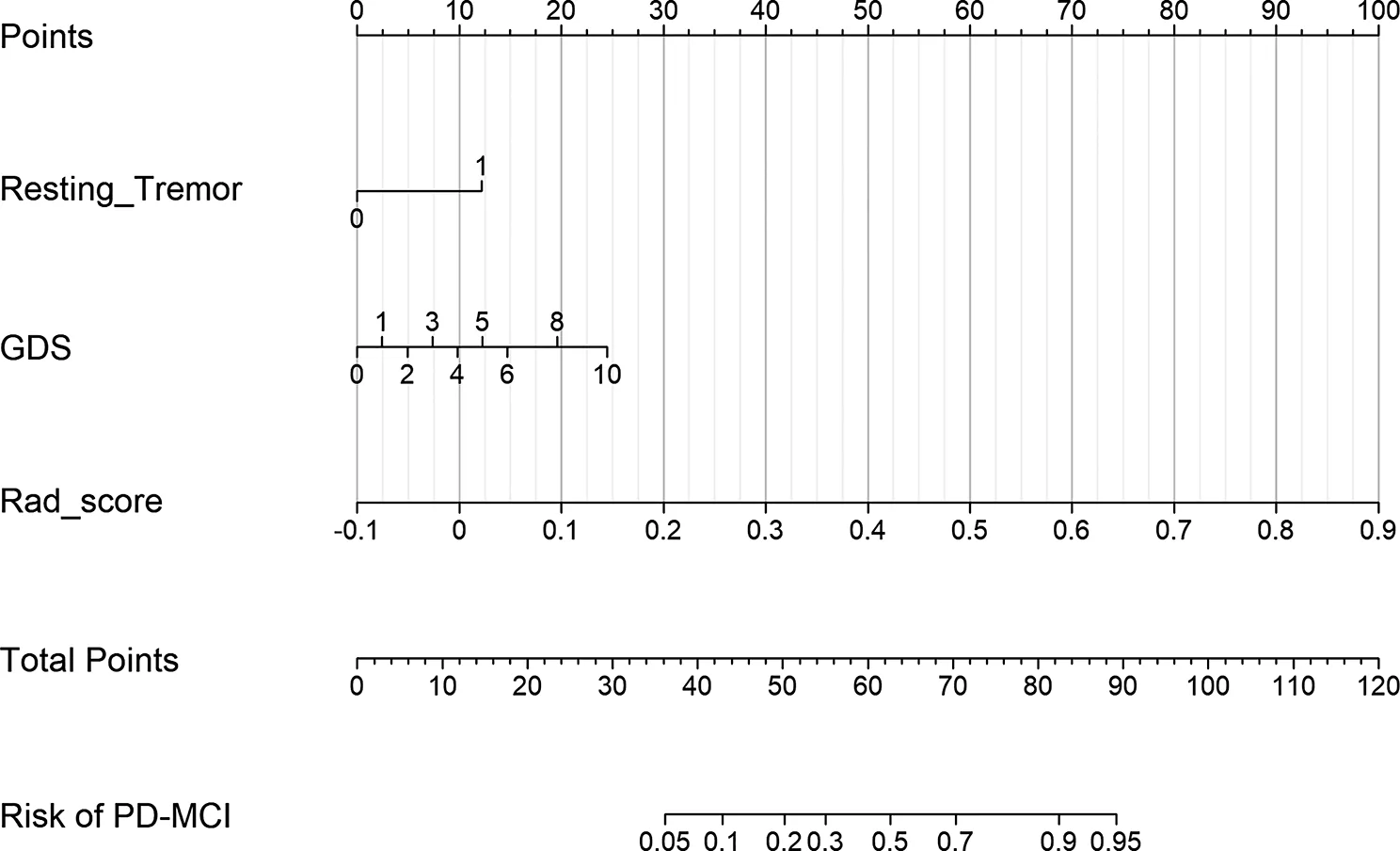
Nomogram for predicting the risk of PD-MCI. To use the nomogram, locate the patient’s values for each predictor on the corresponding axes (Rad-score, GDS score, Resting Tremor), draw a vertical line up to the “Points” axis to determine the points for each, sum the points, and draw a vertical line down from the “Total Points” axis to the “Linear Predictor” and “Risk of PD-MCI” axes to obtain the predicted probability

**Fig. 5:**
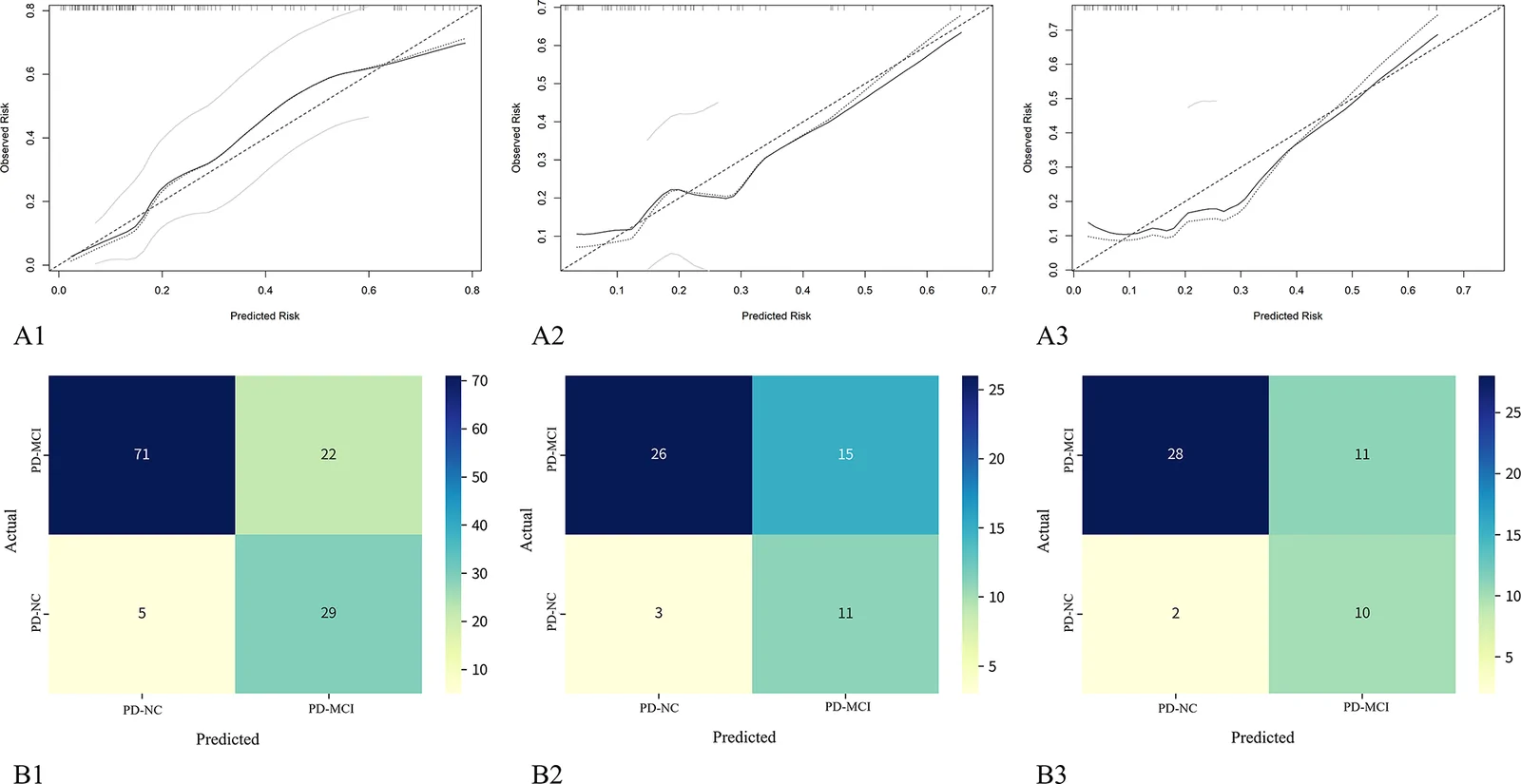
Calibration curves and confusion matrices of the combined model. A1, A2, and A3 show the calibration curves of the combined model in the training, test, and external validation sets, respectively. B1, B2, and B3 show the confusion matrices of the combined model in the training, test, and external validation sets, respectively

**Fig. 6:**
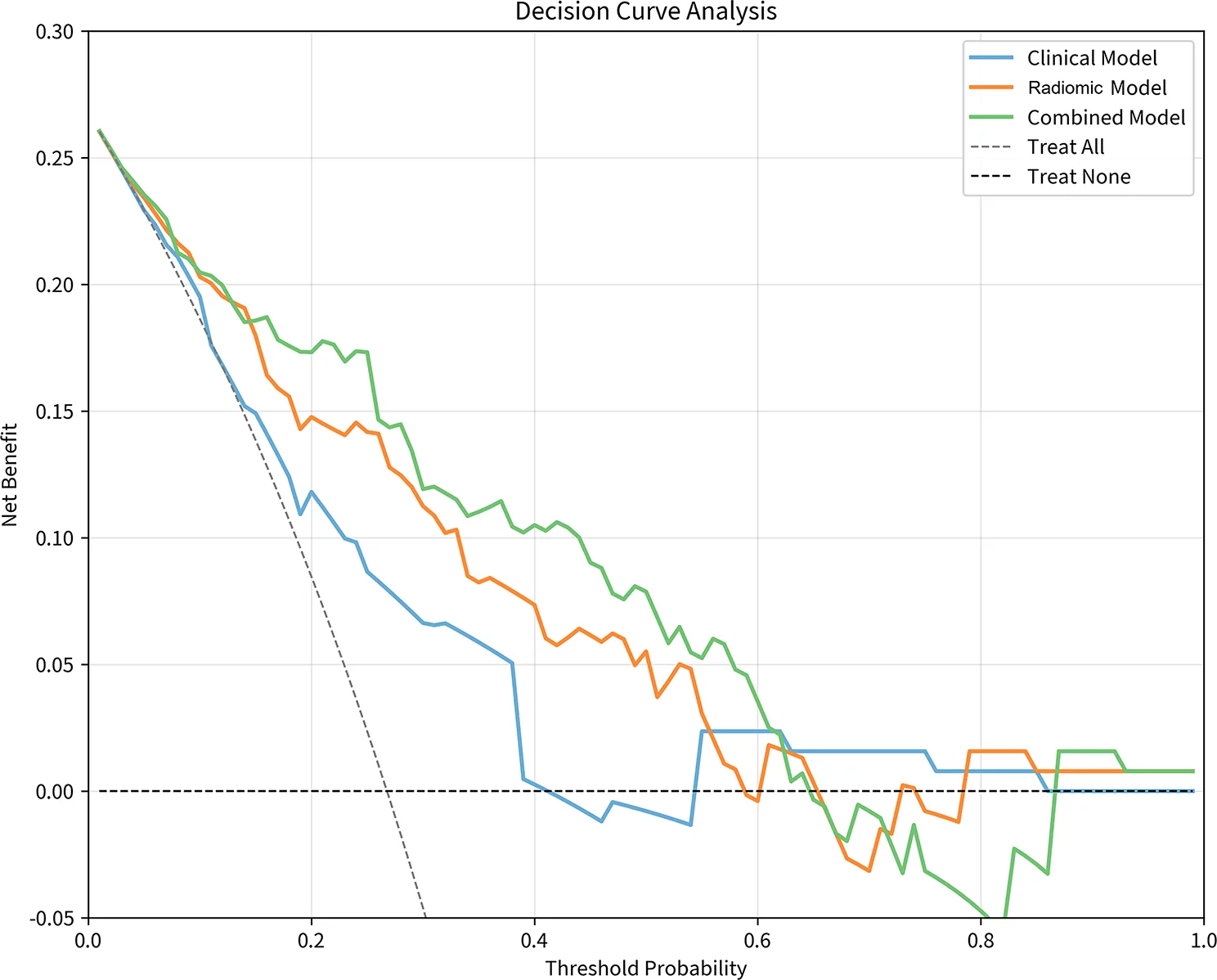
DCA for the clinical model, radiomics model, and combined model

Univariate logistic regression identified resting tremor (odds ratio [OR] = 3.40; 95% CI: 0.95–12.15; *P* = 0.060) and GDS score (OR = 1.36; 95% CI: 1.09–1.70; *P* = 0.007) as potential predictors. Multivariate logistic regression confirmed their independent associations with PD-MCI progression (Table 2). Integration of the Rad-score with these clinical predictors significantly improved model performance. The formula for calculating the combined model is provided in Fig. S2 of the Supplementary Material. The combined model yielded AUCs of 0.848 (95% CI: 0.774–0.906) and 0.796 (95% CI: 0.666–0.893) in the training and test sets, respectively. In the external validation set (*n* = 51, 12 progressors), the AUC was 0.812 (95% CI: 0.628–0.925) (Table 3; Fig. 3). Given the limited event count, bootstrap resampling (1,000 iterations) was performed to assess stability. The bias-corrected AUC remained 0.812, with a bootstrap mean of 0.819 ± 0.075 (95% CI: 0.657–0.949), supporting the robustness of the point estimate despite the wide interval (Fig. S3). To translate this model into clinical practice, a Nomogram was constructed (Fig. 4). This tool enables the estimation of PD-MCI risk by assigning points to each predictor: the Rad-score, GDS score, and resting tremor. The total points are then mapped to a predicted probability. The Hosmer-Lemeshow test showed good model fit (*P* > 0.05), and calibration curves demonstrated excellent agreement between predicted probabilities and actual observations (Fig. 5A). Regarding discriminative performance, confusion matrix analysis (Fig. 5B) yielded F1-scores of 0.682, 0.550, and 0.606 for the training, test, and external validation sets, respectively. Decision curve analysis (DCA) indicated that the combined model yielded greater net benefit than the radiomics-only or clinical-only models across most threshold probabilities (Fig. 6).

**Table 2:** Screening of independent predictive factors

Characteristic	Univariable			Multivariable		
	OR(95% CI)		*P* Value	OR(95% CI)	*P* Value	
Age (years)	1.05 (0.99–1.11)	0.078		NA		NA
Sex	0.72 (0.31–1.69)	0.455		NA		NA
Educyrs	1.01 (0.88–1.16)	0.877		NA		NA
Resting Tremor	3.40 (0.95–12.15)	0.060		4.11(1.10-15.37)		0.036
Rigidity	1.50 (0.58–3.86)	0.404		NA		NA
Bradykinesia	0.74 (0.27–2.01)	0.558		NA		NA
Postural Instability	2.28 (0.73–7.13)	0.158		NA		NA
Other	0.56 (0.17–1.78)	0.322		NA		NA
H-Y	1.89 (0.85–4.20)	0.117		NA		NA
ESS	0.55 (0.15–2.03)	0.367		NA		NA
GDS	1.36 (1.09–1.70)	0.007		1.41(1.11–1.78)		0.005
RBD	1.60 (0.70–3.65)	0.268		NA		NA

Note. Data in parentheses are 95% CI. CI = confidence interval, OR = odds ratio

**Table 3:** Diagnostic performance comparison of the combined model, rad-score model, and clinical model across the training, test, and validation sets

Cohort	Characteristics	AUC	Sensitivity	Specificity	PPV	NPV	*P* value
Training set	Combined model	0.848	0.853	0.763	0.569	0.934	NA
	Rad-score model	0.809	0.941	0.430	0.377	0.952	0.119^a^
	Clinical model	0.698	0.09	0.957	0.429	0.742	< 0.001^b^
Test set	Combined model	0.796	0.786	0.634	0.423	0.897	NA
	Rad-score model	0.777	0.857	0.512	0.375	0.913	0.721^a^
	Clinical model	0.638	0.143	0.902	0.333	0.755	0.021^b^
External validation set	Combined model	0.812	0.833	0.718	0.476	0.933	NA
	Rad-score model	0.776	0.833	0.462	0.323	0.900	0.590^a^
	Clinical model	0.624	0.080	1.000	1.000	0.780	0.030^b^

Note: The markers a and b represent the diagnostic performance comparison of Combined model with the Rad-score model and Clinical model, respectively. NA, not available

### Model interpretability with SHAP analysis

**Fig. 7:**
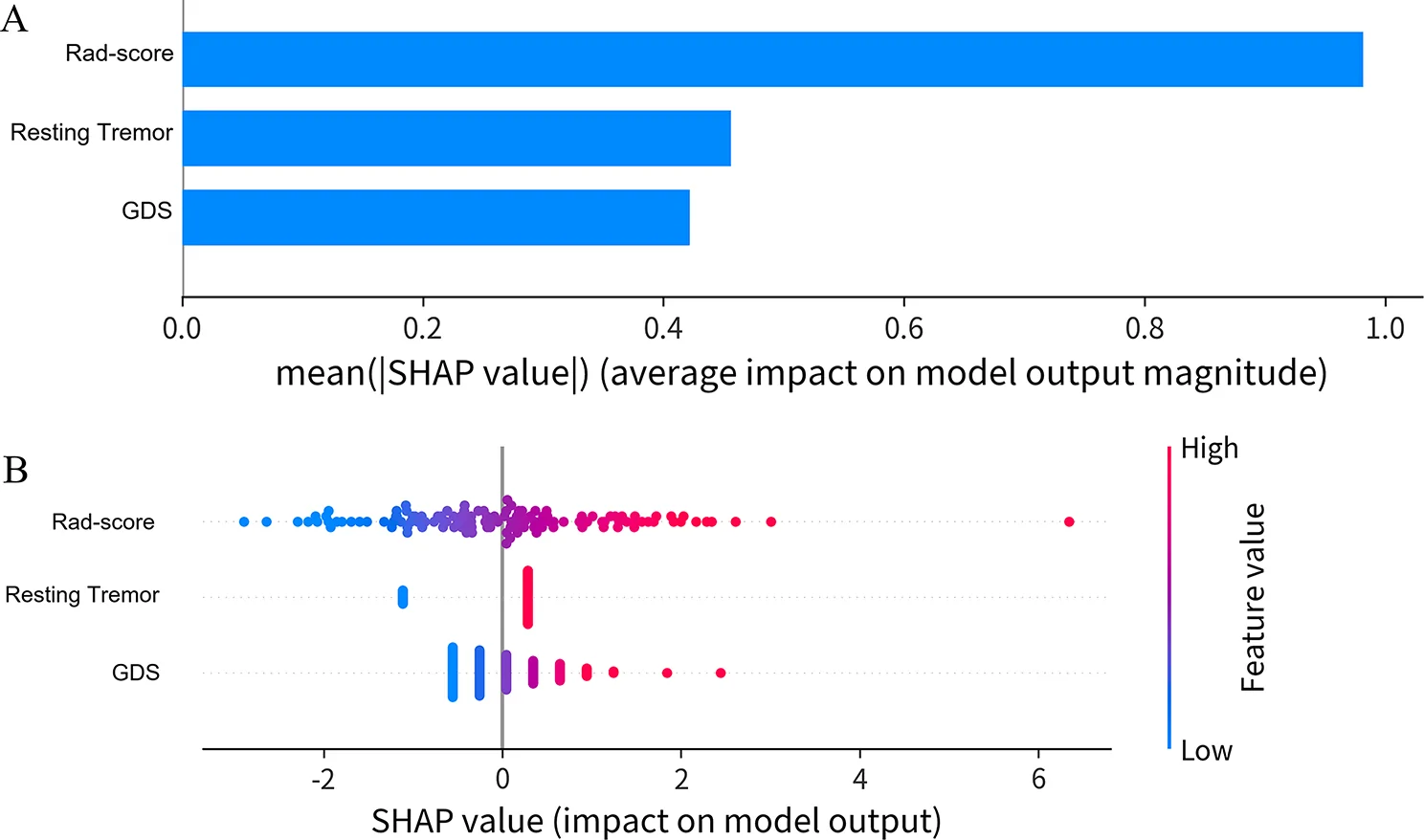
SHAP analysis of the combined model. (**A**) Mean SHAP values of each feature show that R-score contributed most to model predictions. (**B**) SHAP scatter plot demonstrates the distribution of feature impacts, with higher Rad-score values linked to higher predicted higher predicted probabilities of future cognitive decline

SHAP interpretability analysis revealed that the Rad-score was the most important feature in the combined model, contributing substantially more to the model output than resting tremor or the GDS score (Fig. 7A). In the SHAP summary plot (Fig. 7B), higher Rad-score values (red) were positively associated with an increased predicted probability of PD-MCI, confirming its dominant role in the model’s decision-making process.

## Discussion

In this study, we moved beyond conventional whole-brain analyses to specifically target the basal ganglia-thalamic circuitry using conventional MRI sequences. This regional focus represents a significant methodological advancement over previous radiomics studies in PD-MCI. By linking radiomic textures (specifically GLCM-derived heterogeneity) to the microstructural integrity of this circuit, our model provides a biologically anchored explanation for cognitive trajectories. This strategy not only improves diagnostic efficiency but also reduces unnecessary costs and time, demonstrating considerable translational value.

The constructed basal ganglia-thalamic radiomic model achieved AUC values of 0.848, 0.796, and 0.812 in the training, test, and external validation sets, respectively, outperforming single-modality models (Table 3, Fig. 3). By linking microstructural alterations within the basal ganglia-thalamic circuitry—a key substrate for PD cognitive impairment [24]—to clinical predictors like GDS and tremor, our study offers a novel non-invasive biomarker. However, from a practical clinical perspective, the performance profile of the Combined model in the external validation cohort warrants careful interpretation. While specificity fluctuated across datasets (0.763 in training, 0.634 in testing, and 0.718 in external validation), the model maintained robust sensitivity (0.833) and a high NPV (0.933). This supports its utility as a ‘rule-out’ tool for low-risk patients. However, the modest PPV (0.476) necessitates caution against false positives; thus, confirmatory assessments are advised before intervening on positive predictions.

To contextualize these findings within the evolving landscape of MRI-based PD-MCI prediction studies, several distinctions warrant emphasis. In our prior whole-brain radiomics study [23], we extracted over 3,300 radiomic features from diffuse gray and white matter compartments. Even after rigorous dimension reduction that retained 8 final features, these signatures remained anchored to mixed gray-white matter heterogeneous tissue pools rather than specific subcortical nuclei, limiting their precise anatomical interpretability. In contrast, our basal ganglia–thalamic circuitry framework bridges these gaps by targeting PD-relevant nuclei with lower feature dimensionality (107/ROI) and distilling the predictive signal into 13 robust features primarily from the TH and PUT. This specificity is clinically relevant; as the central hub of the somato-cognitive action network (SCAN) [24], the TH serves as the primary relay station for cognitive information, while the PUT represents the earliest site of dopaminergic denervation. Furthermore, unlike many single-center reports, our PPMI (multicentre) + NACC (external) validation design provides a more stringent test of generalizability. While contemporary pipelines often extract > 800 features/ROI via wavelet decompositions (IBSI-standardized), our current extraction prioritizes regional specificity over feature-space breadth, representing a deliberate trade-off between depth and deployability.

With regard to regional specificity, the TH and PUT were identified as the primary contributors within our radiomics model. Specifically, TH-associated features accounted for the most substantial fraction of the selected signature (38.5%, 5/13). Notably, the feature original_shape_Maximum2DDiameterColumnwithin the PUT exhibited the highest coefficient weight (-0.0611). Mechanistically, these findings align with the core pathophysiology of PD. As a pivotal node integrating motor and cognitive information, the TH is a hallmark of PD disruption [30]. Furthermore, as the principal target of nigrostriatal degeneration, the PUT is the primary site of early dopaminergic loss [31]. Consequently, the salient radiomic signatures extracted from these regions effectively capture the underlying structural remodeling associated with PD progression.

Delving deeper into the imaging phenotype, texture features (9 features) and shape features (3 features) constituted the majority of the predictive model. Notably, GLCM-derived features were prominent, accounting for 38.5% (5/13) of all retained features. As a cornerstone of texture analysis, GLCM captures spatial relationships between voxels (e.g., intensity correlation, contrast), quantifying the homogeneity of tissue microstructure [32]. Alterations in these features likely reflect tissue disorganization (e.g., neuronal disarray) and gliosis (e.g., astrocytic proliferation) secondary to basal ganglia circuitry dysfunction. Such pathological changes disrupt tissue uniformity, leading to abnormal spatial distribution of voxel intensities on T1WI, which is precisely captured by GLCM metrics. Supporting this, Chang Li et al. [33] demonstrated that GLCM features derived from the basal ganglia are pivotal for PD classification, while Sivaranjini et al. [34] reported that 3D GLCM features significantly differentiated cognitive impairment statuses. While conventional MRI cannot directly visualize core PD pathologies like α-synuclein aggregation [35, 36], radiomic features indirectly reflect secondary alterations arising from these pathological processes by quantifying microstructural heterogeneity.

To translate these imaging phenotypes into clinical decision-making, we further employed SHAP analysis to interpret the model’s predictions. SHAP unequivocally identified the Rad-score as the most influential predictor, surpassing resting tremor and the GDS score. The SHAP summary plot demonstrated a positive association between higher Rad-score values and increased predicted probability of PD-MCI, underscoring the dominant role of radiomics. Crucially, the high weight of the PUT shape feature and the prominence of TH texture features within the Rad-score provide a biologically grounded explanation for this dominance. The integration of these microstructurally sensitive radiomic features with clinical markers of depression (GDS) and motor severity (resting tremor) creates a comprehensive signature that captures both the subcortical structural substrate and the clinical phenotype of cognitive decline. The identified clinical predictors are also biologically plausible; for instance, the GDS score correlates with CSF p-tau levels, suggesting depression serves as an early warning signal [37]. Although resting tremor exhibits lower specificity for PD-MCI [38], its integration with radiomics compensates for the limitations of single modalities, aligning with established strategies combining striatal radiomics with clinical parameters [38] and reinforcing our model’s validity.

Our study has limitations. First, the retrospective design may have introduced selection bias; thus, prospective, longitudinal, multicenter cohorts are needed to validate the model’s generalizability across diverse real-world clinical settings. Second, the NACC dataset lacked several clinical variables available in PPMI (e.g., rigidity, Hoehn & Yahr stage). Although this limitation does not compromise the validation of our specific combined model, the absence of these variables precludes a comprehensive assessment of motor severity subtypes and disease staging in the external cohort. Future prospective studies should aim to standardize and collect a broader panel of clinical parameters across multiple centers to enable more granular subgroup analyses. Third, the definition of PD-MCI was based on an education-adjusted MoCA score ≤ 25, which represents a Level I screening tool rather than the MDS Task Force Level II criteria—the reference standard requiring comprehensive multi-domain neuropsychological testing [39]. Although this approach is consistent with the PPMI protocol [40] and prior PD radiomics work [41], and included an education-adjustment rule (adding 1 point for ≤ 12 years of education) [27] to mitigate bias across sites, it may introduce misclassification bias and precludes analysis of domain-specific cognitive deficits. Future prospective studies employing full MDS criteria are warranted. Fourth, although PPMI and NACC are multi-vendor, multi-scanner cohorts, we implemented standardized preprocessing. Notably, post-hoc harmonization (e.g., ComBat, WhiteStripe) was not applied. To mitigate potential batch effects, we deliberately omitted wavelet-derived textures, which are particularly susceptible to inter-vendor intensity variations. Future work should integrate rigorous harmonization techniques alongside wavelet features, employ 3D-CNN algorithms for spatial context extraction, and incorporate multi-omics data (e.g., metabolomics) to construct robust multi-scale models. thereby enhancing predictive performance. Notably, while our DCA suggested potential clinical utility, this net benefit requires prospective validation in large-scale, multicenter cohorts to confirm its value in guiding real-world clinical decision-making for PD-MCI management.

## Conclusions

This study systematically develops and validates the predictive value of basal ganglia-thalamic radiomic features for PD-MCI. By integrating high-dimensional imaging features with clinical parameters, the combined model achieved robust predictive performance. Furthermore, model interpretability based on SHAP enhanced its clinical utility, providing a feasible strategy for early risk stratification in Parkinson’s disease.

## Supplementary Information

Below is the link to the electronic supplementary material.

Supplementary Material 1

## Data Availability

Data for this study were obtained from the Parkinson’s Progression Markers Initiative (PPMI; https://www.ppmi-info.org) and the National Alzheimer’s Coordinating Center (NACC; https://naccdata.org). All data were publicly available from these databases.

## Abbreviations

AAL3: Automated Anatomical Labeling Atlas 3
AUC: Area Under the Receiver Operating Characteristic Curve
DCA: Decision Curve Analysis
DTI: Diffusion Tensor Imaging
GP: Globus Pallidus
IBSI: Image Biomarker Standardization Initiative
IRB: Institutional Review Board
LASSO: Least Absolute Shrinkage and Selection Operator
MoCA: Montreal Cognitive Assessment
mRMR: Minimum Redundancy Maximum Relevance
MRI: Magnetic Resonance Imaging
NM-MRI: Neuromelanin-Sensitive MRI
PD: Parkinson’s Disease
PD-D: Parkinson’s Disease Dementia
PD-MCI: PD with Mild Cognitive Impairment
PD-NC: PD with Normal Cognition
PET: Positron Emission Tomography
PUT: Putamen
Rad-score: Radiomics Score
ROC: Receiver Operating Characteristic
ROI: Regions of Interest
SHAP: SHapley Additive exPlanations
sMRI: Conventional Structural MRI
TH: Thalamus
